# Influence of drying temperature on the properties of Colombian banana fibers for its potential use as reinforcement in composite materials

**DOI:** 10.1038/s41598-024-76460-4

**Published:** 2024-10-24

**Authors:** Julián Montoya Berrio, Juan Negrete Martínez, Juan Altamiranda Suárez, César Ávila Díaz, Oswaldo Rivero-Romero, Jimy Unfried-Silgado

**Affiliations:** 1 Department of Mechanical Engineering, University of Cordoba, Montería, Córdoba, Colombia; 2 Department of Mechanical Engineering, University of Antioquia, Medellín, Antioquia Colombia

**Keywords:** Drying temperature, Banana pseudostem fiber, Characterization of fiber, Mechanical properties, Mechanical engineering, Characterization and analytical techniques

## Abstract

This study investigated the impact of drying temperature on the physicochemical and mechanical properties of banana pseudostem fibers sourced from the Cordoba region in Colombia. Banana fibers (BFs) were extracted through mechanical decortication from the banana pseudostem (BP) of the plant and subsequently oven-dried at temperatures of 40 °C and 90 °C. Six mathematical models were employed to analyze the drying behavior of the fibers. The density of the BFs was determined using the apparent density method, and their chemical composition was evaluated via bromatological analysis. Fiber diameter was measured using optical microscopy (OM). The BF samples were characterized by scanning electron microscopy (SEM), Fourier transform infrared spectroscopy (FTIR), thermogravimetric analysis (TG), contact angle measurements, and tensile testing. The results indicated that noncellulosic materials were removed from the fibers when dried at 90 °C, as evidenced by alterations in thermal degradation and fiber surface morphology observed through TG and SEM, suggesting a reduction in lignin content. While drying temperature did not affect fiber stiffness or ductility, a correlation with fiber diameter was noted. Thinner fibers, ranging from 148 to 250 μm, exhibited increased tensile strength and Young’s modulus, attributed to a more compact microfibril arrangement.

## Introduction

Lignocellulosic fibers (LF) have increasingly been applied in polymeric matrix composites as potential replacements for synthetic fibers because of their biodegradability and eco-friendliness^1^. Characteristics such as low density, acceptable specific mechanical properties, high abundance, and low cost are of great interest^1–3^. Nevertheless, the use of LFs for the reinforcement of polymer materials can present limitations due to their hydrophilic nature^4^. Therefore, it may be necessary to implement pretreatments for preservation. Drying is a common practice in LF processing to prevent postharvest deterioration^5,6^. The drying process facilitates the reduction in the water content present in the LF by controlling the temperature. This is important because moisture has a negative effect on the mechanical properties of their^7^. Research has revealed that retaining water in the LF can affect the fiber‒matrix interface properties during composite fabrication through exposure to high temperatures^8^. This can lead to the formation of defects within composites, such as pores, due to moisture evaporation. This moisture includes both free water and bound water^9^. Therefore, studies on the drying process of LF have aimed to determine the optimal drying temperature that allows for improved physical, thermal, and mechanical properties^7^.

Environmental awareness and sustainable development have driven efforts to find alternatives in agro-industrial waste management^10,11^. Currently, the 3R strategy (reduce, reuse, recycle) has promoted the use of these residues in many engineering applications as an effective waste management strategy^12^. In this context, crop waste, such as pseudostem bananas, has been used as a source for extracting LF^13^. Bananas have proven important to the global economy and agriculture, with production volumes increasing in recent decades^14,15^. Colombia has a significant banana production area within its territory; however, it is estimated that 70% of the final production is generally considered waste after harvesting due to a lack of management^16,17^. Thus, banana fiber (BF) has been investigated for applications in manufacturing engineered materials^18,19^. Studies have reported that BFs have a good strength-to-weight ratio, making them excellent candidates for the development of lightweight composites^20,21^. Therefore, within the Colombian agroindustry, there is a significant opportunity to transform and utilize BP as a source of BF to produce polymeric composites^16^.

The drying process is a simple and low-cost pretreatment that prevents deterioration and extends the shelf-life of BFs^22^. Nevertheless, excessive drying temperatures or extended drying times can cause morphological changes and structural damage in BFs. Research reveals that temperature and moisture gradients produced during drying can cause shrinkage of LF^5,11^. Additionally, changes in the temperature of the fiber can generate thermomechanical stresses that cause the fiber to weaken and, which in turn, contribute to the loss of adhesion between the fiber and the matrix^23^. Therefore, determining the ideal temperature is an important step in the BF drying process, as it can guide researchers in their future work in composite manufacturing^22^. According to Martinelli et al.^7^, drying LF at 70 °C may provide fiber-reinforced composites with high ductility and tensile strength. On the other hand, Oliveira et al.^5^ conducted an experimental study on the drying kinetics of BF at drying temperatures of 60, 75, and 90 °C. Their results revealed that the diffusion coefficient increased with increasing drying temperature, indicating greater diffusion of moisture into the fibers at higher temperatures. However, few studies have determined the effects of lower drying temperatures on BFs for potential use in composites. This latter is important because, at higher drying temperatures, the final energy consumption is greater^22^. Furthermore, identifying drying temperatures that produce LFs with increased strength could lead to reductions in both costs and energy consumption. This is essential for developing environmentally sustainable materials for industrial applications^24^.

The goal of this work was to evaluate the influence of drying temperature on the properties of Colombian banana pseudostem fibers. The banana fibers were oven-dried at 40 °C and 90 °C. The drying behavior of the fibers was analyzed via six mathematical models. The apparent density technique was used to calculate the density of BFs, and bromatological analysis was used to assess their chemical composition. Both samples were characterized via microscopic optical microscopy, scanning electron microscopy, Fourier transform infrared spectroscopy, thermogravimetric analysis, contact angle measurement, and tensile tests. A comparative analysis between the two samples was conducted.

## Materials and methods

### Materials

**Fig. 1:**
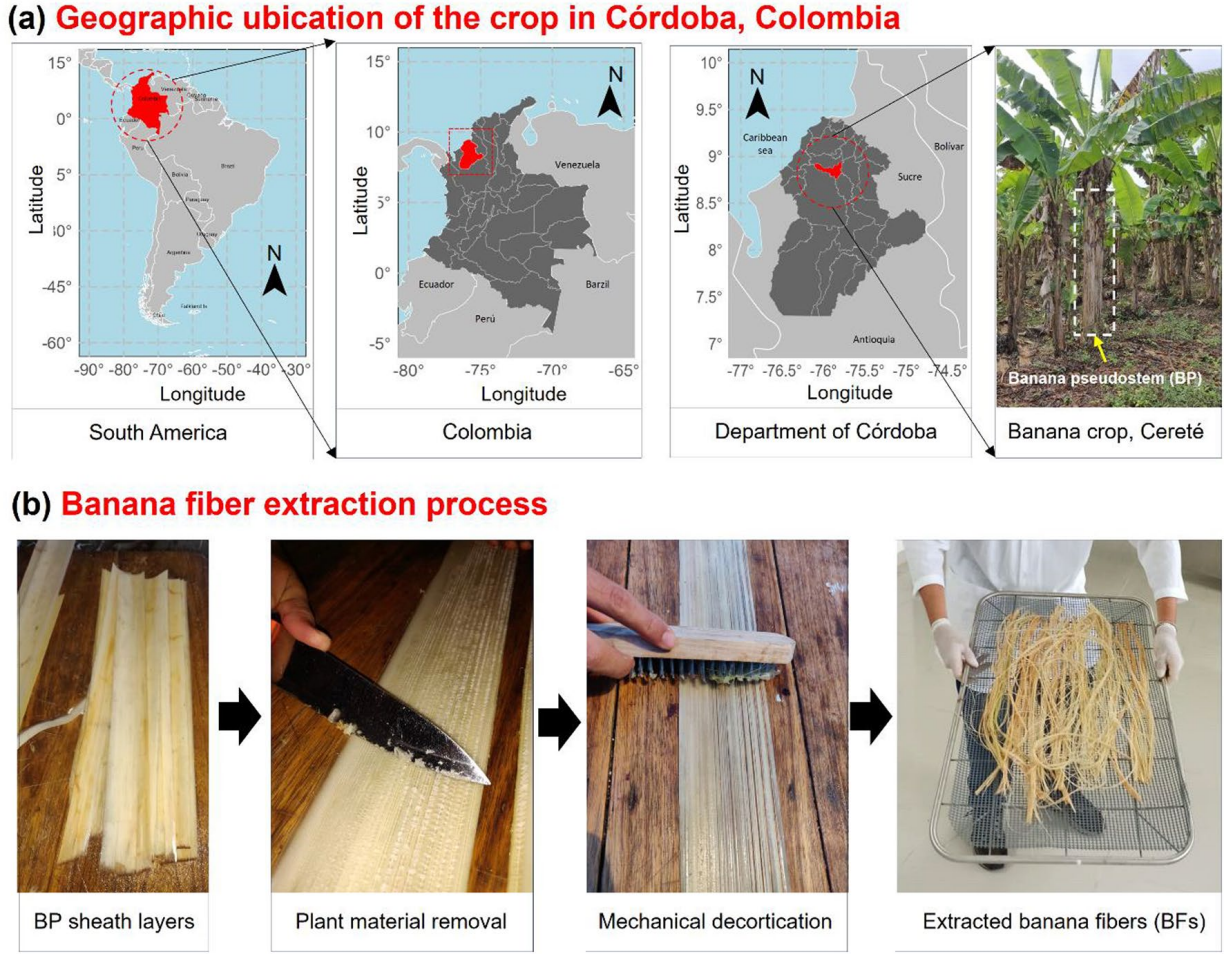
(**a**) Geographic location of banana crops in Córdoba, Colombia, using R (version 4.2.2, https://www.r-project.org/), and (**b**) banana fiber extraction process employed.

In this study, BFs were extracted from the pseudostem of the plant *Musa paradisiaca*, which normally grows in the warm and temperate areas of Colombia^25^. *Musa paradisiaca* belongs to the family Musaceae, and the species was first formally described by Caroli Linnaeus in 1753^26^. The plant sample has been cataloged with the register number COAH/8066 by Clara Henao, Colombian Amazon Herbarium, Amazon Institute of Scientific Research-SINCHI^27^. Additionally, a voucher specimen is preserved at the University of Córdoba Herbarium with catalog number HUC 9779. The BPs were collected from the same crop in the municipality of Cereté, Córdoba, Colombia (12 MASL), located at coordinates 8°51’33” N, 75°49’43” W. Figure 1(a) shows the geographic ubication of the crop in Córdoba, Colombia. The maps were generated using open-source R software (version 4.2.2, https://www.r-project.org/) within RStudio (version 2022.12.0.353, https://posit.co/download/rstudio/) through the following packages: rnaturalearth (version 1.0.0, https://docs.ropensci.org/rnaturalearth/), sf (version 1.0–17, https://r-spatial.github.io/sf/), and ggplot2 (version 3.4.2, https://ggplot2.tidyverse.org/). Since the BFs are a by-product of the harvested banana crop, no special permissions were required for their extraction and study. The BF extraction process was carried out as shown in Fig. 1(b). First, the BP sheath layers were separated and cut to a length of 0.5 m. Then, the plant material was manually removed with a sharp surface. Finally, the BF was extracted to simulate the mechanical decortication process via a metal brush, and sufficient force was applied to remove the remaining plant material and accumulated water. The BFs were stored in plastic bags to preserve their extraction conditions.

### Drying procedure

The extracted BFs were dried at two different drying temperatures, 40 °C and 90 °C, following the procedure of the ASTM 4442-20 standard for moisture content sampling. A total mass of 250 g of wet BF was weighed for both drying treatments via an Adventurer analytical balance model ARC120 with a sensitivity of two significant figures. BPG-9070 A drying oven model I with a temperature range of 10 °C to 200 °C, a hot air velocity of 1 m/s, and an error margin of ± 1 °C was used for the drying processes. The oven was preheated to the required temperature for each drying temperature before placing the sample inside. Then, the fibers were distributed in a drying tray to take advantage of the forced air circulation of the oven. Drying curves were determined experimentally through periodic sampling of moisture content^28^. The samples were removed from the oven, and their moss loss was continuously measured at 10-, 15-, and 30-minute intervals until the mass loss was equal to or less than 1% of the previously measured value. Equation (1) was used to calculate the MC.

1 $$MC (\%)=\frac{{W}_{0}-{W}_{t}}{{W}_{t}}\times 100\%$$

where *W*_*t*_ – represents the oven-dry mass at time t (g), and *W*_*0*_ – represents the original mass (g).

### Modeling of the drying curve

Six mathematical models were adjusted to predict the drying behavior of the samples at two drying temperatures to validate the experimental drying curves of the BF^5,29^. Table 1 summarizes the mathematical models used. Adjustments were made via nonlinear regression (Polymath Professional 6.10). The best-fitting model under two different conditions was selected based on several statistical criteria, including the coefficient of determination (R^2^), the adjusted coefficient of determination (R^2^_adj_), the Chi-square (χ^2^), and the root mean square error (RMSE)^5^. Higher values of the coefficient of determination and lower values of the root mean square error and Chi-square are preferred for the fitted models^30^.

**Table 1:** Mathematical drying models.

No.	Model name	Model equation	Refs.
1	Lewis	$y=exp(-kt)$	Elfaleh et al.^4^
2	Page	$y=exp(-k{t}^{n})$	Alomayri et al.^3^; Elfaleh et al.^4^
3	Modified Page	$y=exp\left({-\left(kt\right)}^{n}\right)$	Alomayri et al.^3^
4	Henderson and Pabis	$y=a\bullet exp(-kt)$	Alomayri et al.^3^; Elfaleh et al.^4^
5	Diffusion approach.	$y=a\bullet exp(-kt)+\left(1-a\right)\bullet exp(-kbt)$	Alomayri et al.^3^
6	Midilli et al.	$y=a\bullet exp(-k{t}^{n})+bt$	Alomayri et al.^3^; Elfaleh et al.^4^

### Fiber characterization

#### Apparent density determination

The apparent density of the BF was determined via the ISO 5311:1992 standard. To calculate the apparent density, the dry weight of the fibers was divided by the observed compacted volume of the sample in a predefined volume container of 508.33 cm^3^. Before measurement, the fibers were oven-dried at 90 °C for 180 min and ground to achieve a uniform particle size. Equation (2) was used for the calculation of the apparent density of the fibers.

2 $${\rho}_{a}=\frac{m}{v}$$

where *m* is the mass of the BFs deposited in the container (g), *v* is the volume of the particles, including internal pores (cm^3^), and *ρ*_*a*_ is the apparent density (g/cm^3^).

#### Bromatological analysis

Bromatological analysis was performed on the oven-dried BFs at 40 °C. The mass percentages of acid detergent fiber (ADF), neutral detergent fiber (NDF), and cellulose were determined via standard methods proposed by AOAC International^31^. The mass percentages of lignin and hemicellulose were determined according to^32^.

#### Diameter measurement

The fiber diameter of the samples dried at 40 °C (BF40) and 90 °C (BF90) was measured via optical image microscopy. Images at 5× magnification were captured via a stereoscopic microscope (Leica EZ4 D) and image acquisition software LAS EZ. The equivalent diameter was measured via transverse measurements along the fiber length^33^. The measurements were taken in 15 distinct regions along the fiber length. Twenty samples were analyzed at each drying temperature.

#### Morphological analysis

The surface morphology of the samples dried at 40 °C and 90 °C was evaluated by scanning electron microscopy (SEM) with a JEOL JSM-7100 F microscope, equipped with a Bruker secondary electron (SE) detector. An accelerating voltage of 10 kV and a working distance (WD) of 20 mm were used. The samples were fixed to a brass mount holder with double-sided carbon adhesive tape. The BFs were coated with a thin gold layer to ensure conductivity via a Quorum model Q300T D sputter coater. Cross-section and longitudinal section images of the samples were captured at 25×, 150×, 300×, and 500× magnification, respectively.

#### Fourier transform infrared spectroscopy

Fourier transform infrared spectroscopy (FTIR) analysis of the samples was conducted using a Thermo Fisher Nicolet Summit X FTIR spectrometer equipped with an Everest Diamond ATR (Attenuated Total Reflectance) accessory. FTIR spectra were collected at a resolution of 4 cm^− 1^ over a wavelength range of 4000–400 cm^− 1^. The peaks were analyzed and compared with those in open-access databases.

#### Contact angle measurement

**Fig. 2:**
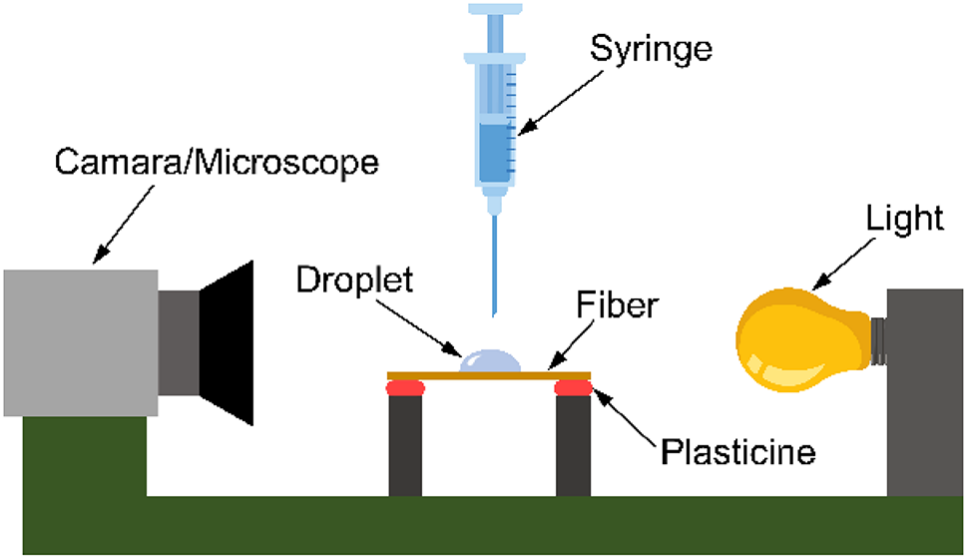
Experimental setup for contact angle measurement^30^.

The contact angle measurement for the BF was carried out following the procedure of the ASTM D7334 standard. Figure 2 depicts the experimental setup implemented for the measurement^34^. An ~ 0.7 µL deionized water droplet size was dropped on the fiber surface to be tested. A digital microscope was used to magnify the contact area, and images were taken within 30 s of drop deposition. Contact angle measurements were made with five drops on a sample for each drying temperature. Two angle measurements (one at each drop edge) were taken from each of the five drops on the surface of the fiber. The average of the ten angles measured was then calculated.

#### Thermogravimetric analysis

BF thermogravimetric analysis (TGA) was performed on a Discovery Q600 thermogravimetric analyzer (New Castle, DE, USA). Approximately 5 mg of each carefully ground sample was weighed and placed in a lead crucible for analysis. The procedure was performed under a nitrogen atmosphere with a heating rate of 10 C/min from 25 to 950 °C, following the ASTM E1131 standard. The weight loss and derivative curve (DTG) were determined and evaluated as a function of temperature.

#### Fiber tensile test

Tensile tests were conducted on individual fibers via a Shimadzu Autograph AG-X universal testing machine equipped with a 500 N load cell. The tests were performed at a testing speed of 5 mm/min and a gauge length of 50 mm according to the ASTM-D3822 standard. Before testing, the ends of individual fibers were carefully glued on a paper frame of 30 × 110 mm using cyanoacrylate adhesive. The samples were conditioned at 25 °C and 50% HR for 24 h. The cross-sectional area of the fibers was calculated through the mean diameter determined by digital image analysis. The tensile properties of the fibers, such as tensile strength, Young’s modulus, and elongation at break, were calculated. Twenty tests were performed at each drying temperature.

## Results and discussion

### Drying curve analysis

**Fig. 3:**
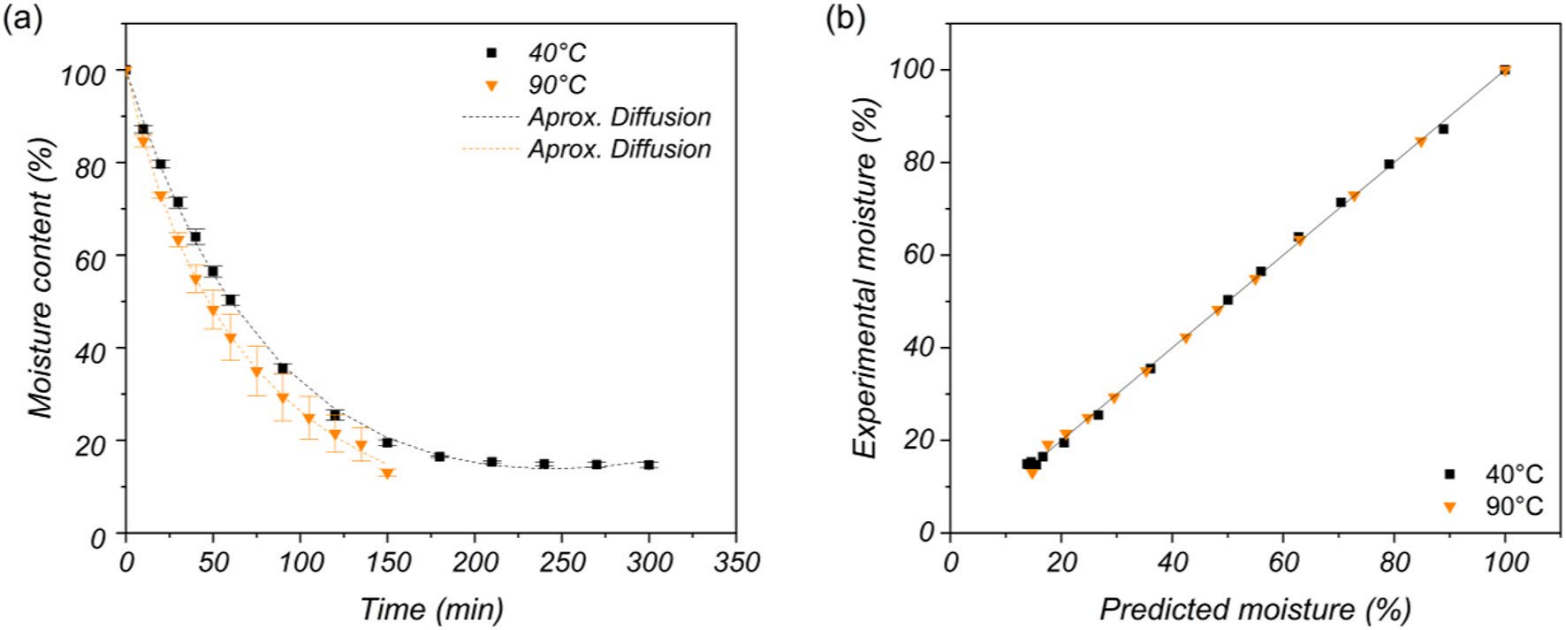
(**a**) Drying curves with the diffusion approximation model fitted to the experimental data and (**b**) comparison of the experimental and predicted values.

Figure 3 presents the results of the drying curve analysis of the BF for both drying temperatures. Figure 3(a) displays the drying curves of the BF samples dried at 40 °C and 90 °C. The experimental drying curves are represented via data points, and the fitting model is shown as a dashed line. The MC decreases with increasing drying time because of the gradual removal of water from the fibers^5^. The MC decreased to 14.69 ± 0.60% at 40 °C for a drying time of 300 min, whereas that at 90 °C was 13.02 ± 0.80% for 150 min. These values are around the ranges reported for dried banana pseudostem fibers by^35,36^. The MC equilibria were reached at different times. The sample dried at 40 °C had a constant moisture content after 200 min, whereas the sample dried at 90 °C reached the final moisture-reducing time at 150 min. A higher moisture removal rate was observed at higher drying temperatures, i.e., 90 °C, because temperature is the main driving force of moisture evaporation^30^. Table 2 summarizes the parameters fitted for the different models and the corresponding statistical analysis results. All the models produced acceptable fitting results. However, the diffusion approximation model was the most suitable for describing the drying kinetics of the studied BF. This model produced R^2^_adj_ values close to 1 and χ^2^ and RMSE values close to zero for both drying temperatures. Therefore, this model best describes the MC curve (see Fig. 3a). This finding is consistent with the results reported by^5^, who concluded that the diffusion approximation model best describes the drying process of banana pseudostem fibers, achieving the highest R^2^_adj_ (0.978) and lowest χ^2^ and RMSE values^5^. The experimental moisture content versus the values predicted by the diffusion approximation model is presented in Fig. 3(b). The linear nature of the curve confirms the validity of the selected model for the drying data of a BF with a 45° slope^22^.

**Table 2:** Parameters and regression coefficients of the different models applied to the drying curves of the BF.

Drying temperature	Models	k	a	b	*n*	*R* ^2^	*R* ^2^_adj_	ERMS	χ^2^
40 °C	1	0.0105	–	–	–	0.9759	0.9759	0.0453	2.20 × 10^− 3^
	2	0.0215	–	–	0.8398	0.9869	0.9859	0.0929	1.29 × 10^− 3^
	3	0.0164	–	–	0.6415	0.9759	0.9741	0.0453	2.37 × 10^− 3^
	4	0.0099	0.9612	–	–	0.9787	0.9771	0.0426	2.09 × 10^− 3^
	5	0.0121	0.9848	-0.5903	–	0.9991	0.9990	0.0087	9.53 × 10^− 5^
	6	0.0088	0.9883	0.0005	1.0829	0.9993	0.9992	0.0075	7.64 × 10^− 5^
90 °C	1	0.0140	–	–	–	0.9938	0.9938	0.0205	4.57 × 10^− 4^
	2	0.0222	–	–	0.8900	0.9992	0.9991	0.0075	6.72 × 10^− 5^
	3	0.0164	–	–	0.6415	0.9938	0.9932	0.0205	4.98 × 10^− 4^
	4	0.0134	0.9698	–	–	0.9959	0.9955	0.0018	3.27 × 10^− 4^
	5	0.0398	0.2021	0.2824	–	0.9993	0.9991	0.0070	6.41 × 10^− 5^
	6	0.0219	1.0019	0.0000	0.8967	0.9992	0.9989	0.0021	8.01 × 10^− 5^

### Density results

In the production of lightweight composites, fiber density is an important property to consider for industrial applications^37^. In this study, the apparent density of the BF was found to be 0.322 g/cm^3^, which is consistent with previous research on fibers extracted from BP^36,38^. Moreover, this value is lower than those of other fibers used for composite reinforcement, such as jute, kenaf, flax, and curaua (0.68–0.78 g/cm^3^)^39^. A lower density is preferred in the production of biocomposites because of its potential for creating lightweight and biodegradable components^32^. However, this value was lower than that obtained by^19^ (0.83 g/cm^3^). The variability in density for the same species of banana plant can vary due to environmental or genetic factors and the age of the individuals^40^.

### Bromatological analysis results

The results of the bromatological analysis of the BF are shown in Table 3. Among the lignocellulosic components, cellulose has the highest percentage value (41.77%), followed by lignin (13.61%) and hemicellulose (11.51%). The high cellulose content is advantageous for composite manufacturing because cellulose has been reported to have high strength and stiffness, consisting of 1,4-linked D-glucose chains arranged in crystalline and amorphous regions^41^. The lignin content of the BF samples was similar to that reported by^19^. The presence of lignin can influence fiber structure and properties by acting as an amorphous adhesive between microfibrils, facilitating the association between cellulose and hemicellulose^42^. In addition, a lower lignin content improves adhesion to polymeric matrices when used in composite manufacturing^19^. In contrast, the hemicellulose content of BF is comparable to that of other bast fibers, such as jute fibers and sisal fibers^43^. The hemicellulose content of these fibers could increase their elasticity^44^. Therefore, on the basis of bromatological analysis, the studied banana fibers are a viable option for reinforcing composite materials.

**Table 3:** Chemical composition of the BFs.

Component	Mass percentage (%)	Used technique
ADF	54.78	AOAC 973.18
NDF	66.29	AOAC 2002.04
Lignin	13.61	Calculation
Cellulose	41.17	AOAC 973.18
Hemicellulose	11.51	Calculation

### Diameter analysis

**Fig. 4:**
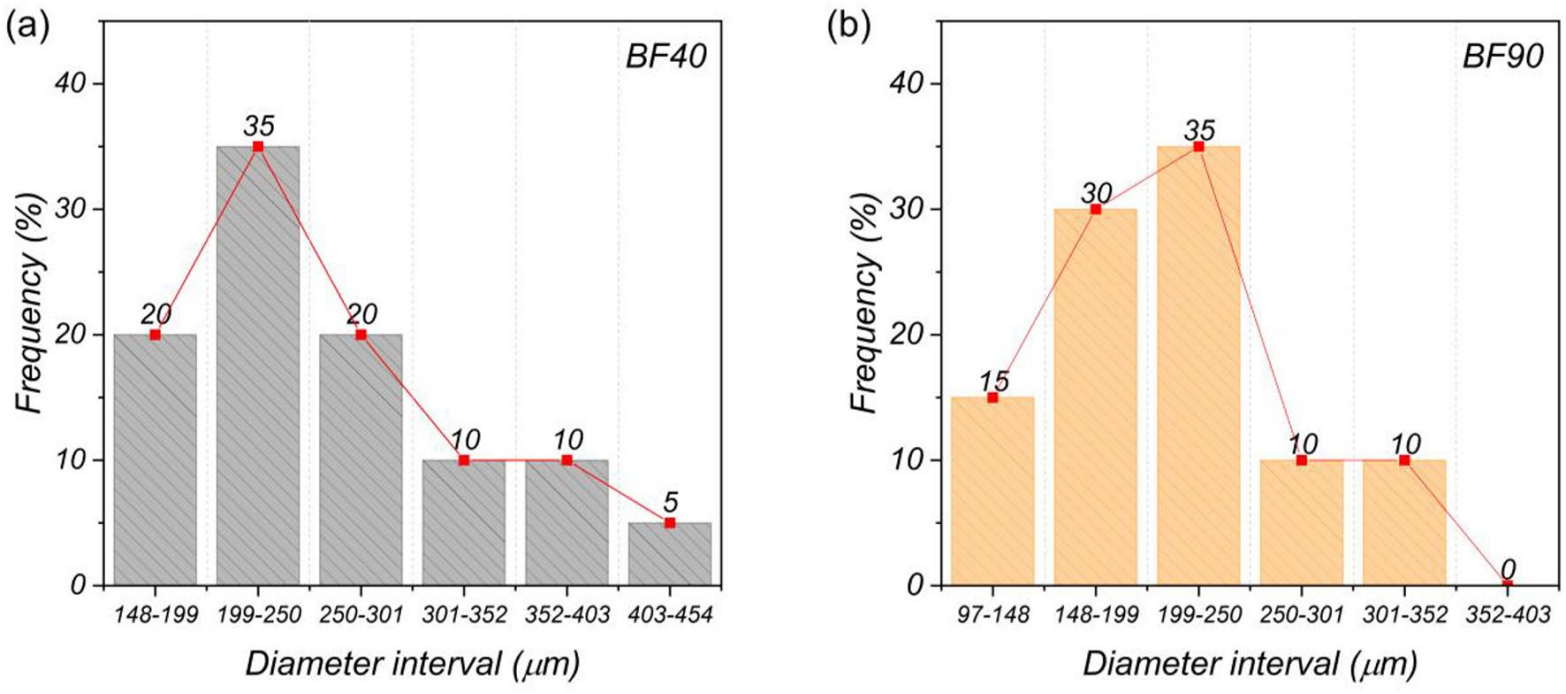
Diameter frequency distributions of (**a**) BF40 and (**b**) BF90.

Table 4 shows the summary statistics of the diameter measurements for the samples heated at 40 °C and 90 °C. The diameter of BF40 ranged from 148.4 to 419.9 μm, with an average fiber diameter of 251.7 ± 73.0 mm. The BF90 had an average fiber diameter of 195.7 ± 58.2 μm, ranging from 96.6 to 319.9 μm. On the other hand, the median value of BF90 is slightly lower than that of the BF40 sample, which may indicate that the diameter of both samples varies. Figure 4 shows the diameter frequency distributions for both BF40 and BF90. As shown in Fig. 4, the BF40 sample contains fibers with diameters up to 454 μm, which is larger than those of the BF90 sample. In addition, a higher frequency (35%) for the sample at 40 °C was obtained in the interval from 199 to 250 μm, followed by the ranges from 148 to 199 μm and 250–301 μm, with values of 20% each (Fig. 4a). In contrast, the BF90 samples had frequency values of 15% between 97 and 148 μm, whereas the samples with diameter values above 250 μm had lower frequency values (see Fig. 4b). The variation in the fiber diameter of the BF was similar to the range reported^45^. Several factors can influence the variation in the diameter of the BF. Factors such as the nature of growth, the age of the plant, and the environmental conditions can influence the heterogeneous characteristics of fiber^33,45^. Similarly, the method of fiber extraction can affect diameter variation. For example, mechanically extracted fibers may retain plant material as a residual binder, leading to fibers with a larger diameter^46^. On the other hand, the tendency of the fiber diameter to decrease at a drying temperature of 90 °C can be attributed to physical changes due to the removal of free and bound water. This can reduce the external volume of a fiber and contribute to the concentration of its chemical constituents^7^.

**Table 4:** Summary statistics of BF diameter in µm.

Sample	Min.	1st Qu.	Median	Mean	3rd Qu.	Max.	sd.
40 °C	148.4	199.3	235.1	251.7	289.9	419.9	73.0
90 °C	96.6	150.9	198.1	195.7	233.3	319.9	58.2

### SEM analysis

**Fig. 5:**
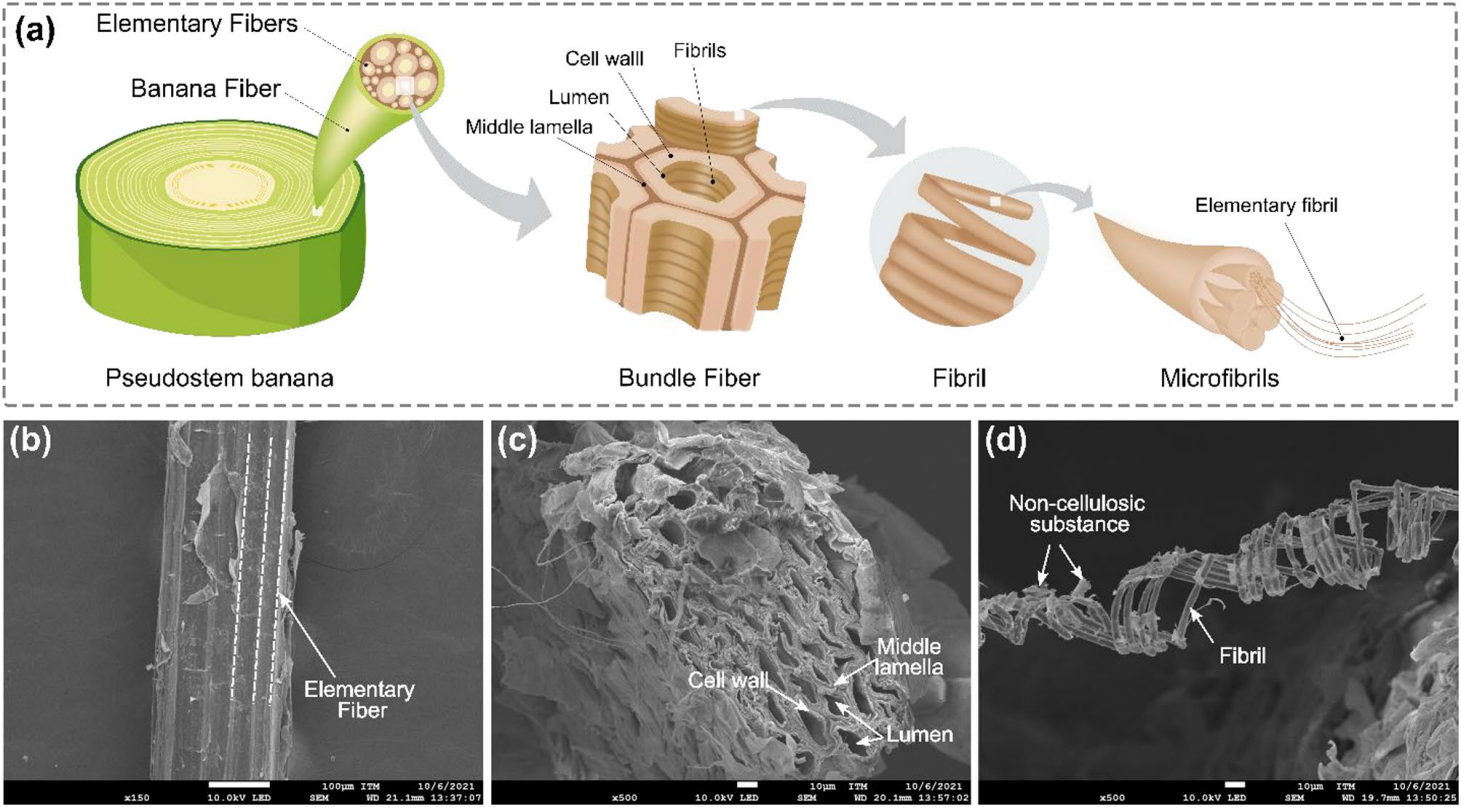
(**a**) Hierarchical structuration of BFs and SEM images of the studied fibers: (**b**) longitudinal section, (**c**) cross-section, and (**d**) banana fibril magnified to 500× (images of the sample dried at 40 °C).

Figure 5(a) shows the hierarchical structuration of the studied BF. These fibers are composed of multiple elementary fibers aligned longitudinally, which are responsible for providing mechanical strength and rigidity (see Fig. 5b)^47,48^. As previously reported^47^, BF has an uneven surface covered by a layer of impurities essentially constituted by hemicellulose and lignin compounds. Additionally, other noncellulosic substances, such as pectin and waxes, are present on the surface, serving as natural binders that remain after mechanical extraction^46^. Figure 5(c) shows the cross-sectional image of the BF. The structure of the BF is characterized by bundle fibers composed of microscope tubes, i.e., elementary fibers, which consist of cell walls surrounding the central lumen and are delimited by a cementation region known as the middle lamella^47,49^. Each elementary fiber consists of helical spirals, also known as fibrils, composed of cellulose microfibrils and noncellulosic components such as hemicellulose and lignin (see Fig. 5d)^50^. This arrangement provides mechanical strength to the BF.

**Fig. 6:**
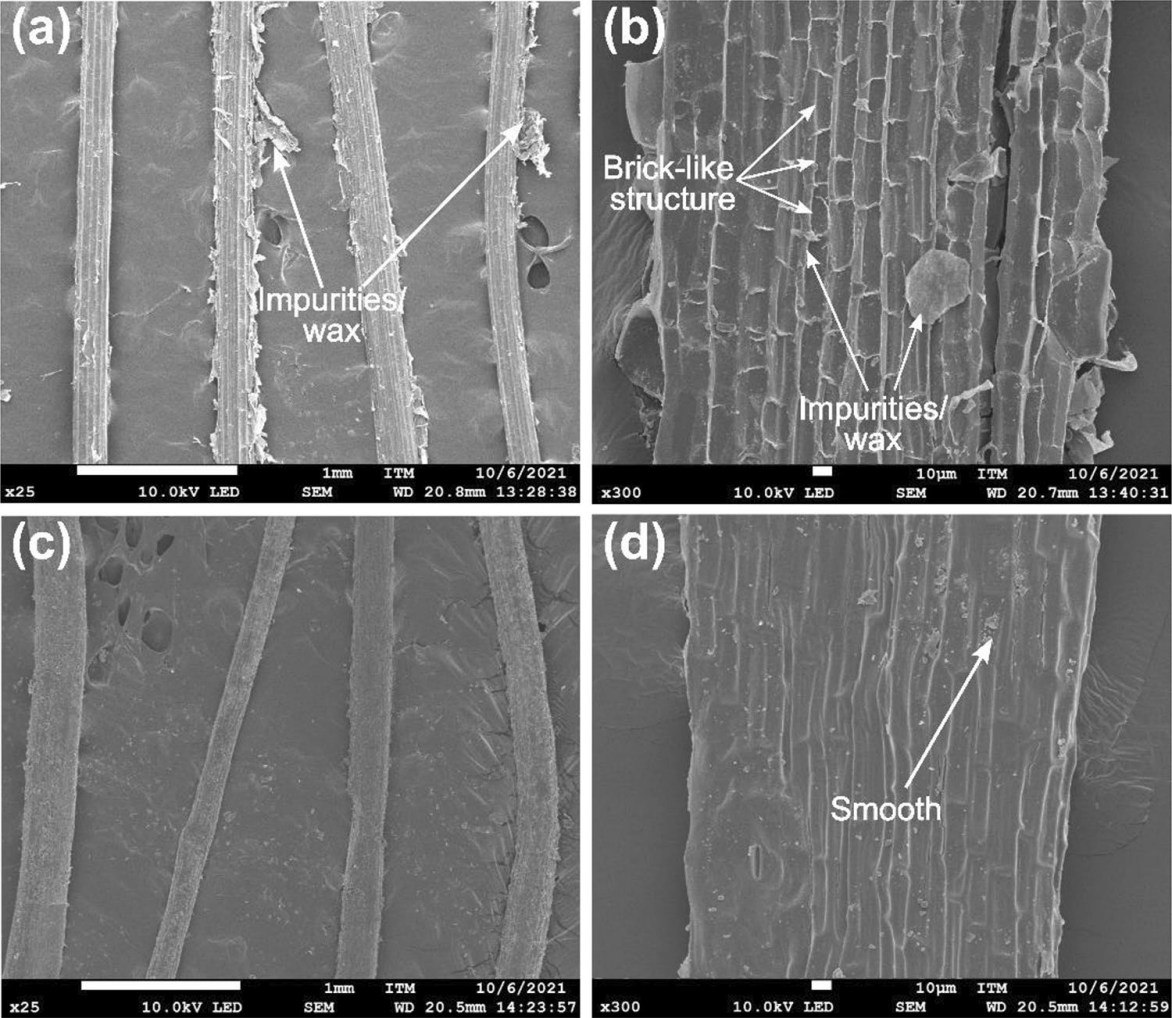
SEM images of the surface morphology of banana fiber samples at different drying temperatures: (**a**) 40 °C, (**b**) BF40 magnified to 300×, (**c**) 90 °C, and (**d**) BF90 magnified to 300×.

The surface morphologies of the dry fibers are displayed in the SEM images in Fig. 6. BF40 involves the presence of impurities, such as waxes and pectin, on the surface^23,47^. These impurities are plant materials retained during mechanical extraction and may remain present after drying at 40 °C^7,46^. In addition, the magnified image (500×) of BF40 revealed a brick-like structure on the fiber surface, which is common for natural fibers^44,51^. This structure comprises parenchymatous cells along with other fiber components, such as lignin, hemicelluloses, and waxes^52^. In contrast, the surface of the fibers changed with increasing drying temperature at 90 °C (see Fig. 6c). BF90 has a surface with fewer impurities, and smooth areas can be observed on the fiber surface, as shown in Fig. 6(d). This may result from the removal of noncellulosic components such as lignin, pectin, and wax during drying at 90 °C^7^. This latter may explain the tendency of the diameter reduction of the BF at this temperature. On the other hand, irreversible changes such as cracking or fibrillation were not observed in the samples after each drying process. This suggests that the loss of moisture and volatiles during the drying of the fibers did not cause partial damage because of the steep temperature and moisture gradients^5^. This is important because the absence of damage to the BF should not affect its mechanical performance as a reinforcement in composite materials.

### FTIR analysis

**Fig. 7:**
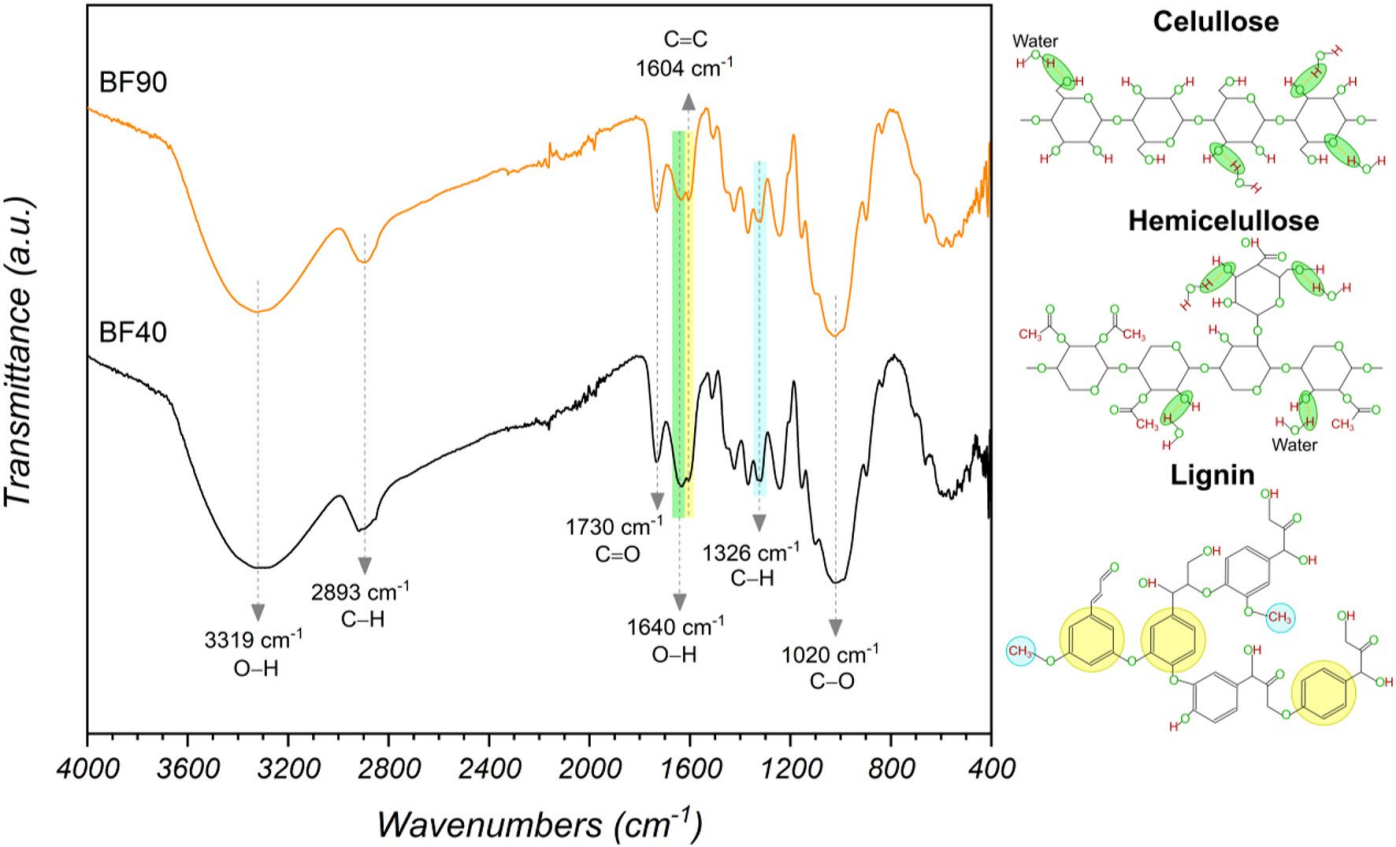
FTIR spectra of samples dried at 40 and 90 °C. Chemical moieties associated with the absorption bands of cellulose, hemicellulose, and lignin.

Figure 7 shows the IR spectra of the dried banana samples in the frequency range between 4000 and 400 cm^− 1^. Absorption bands, which are typical vibrational bands corresponding to cellulose, hemicellulose, and lignin, were observed. These findings are consistent with the results of the bromatological analysis presented in Table 3, where cellulose was found to be the main fiber constituent, followed by lignin and hemicelluloses. The broad absorption band at 3319 cm^− 1^ in both spectra is assigned to the stretching vibrations of the hydroxyl group (O-H) of cellulose, hemicellulose, and lignin^53^. Bands at 2893 cm^− 1^ have been attributed to the stretching vibrations of C-H groups present in its three major constituents^54,55^. The band at 1730 cm^− 1^ was attributed to the stretching vibration of the acetyl group in hemicelluloses. The peaks at approximately 1640 cm^− 1^ and 1604 cm^− 1^ were associated with O–H bending due to the presence of water in the fibers and the aromatic skeletal vibration of lignin^56,57^. The band at approximately 1326 cm^− 1^ was assigned to the C-H vibration and stretching of phenolic OH groups, which are characteristic of the lignin structure^58^. The peak at approximately 1030 cm^− 1^ was due to the C-O stretching vibration in cellulose and lignin^57^. All the above bands were present in both FTIR spectra. There were no significant differences in the FTIR spectra of the samples. In general, drying did not result in displacement or disappearance of absorption bands because degradation of hemicellulose and cellulose in the pseudostem fibers occurred only at temperatures above 190 °C^5^. However, some minor differences were observed between BF40 and BF90, suggesting changes in the chemical composition during the drying process. For example, the most intense band at 1640 cm^− 1^ was observed in BF40, which may indicate a greater presence of water in the banana fibers after drying at 40 °C. This may be because water is more easily retained in the cell wall of the fiber at a drying temperature of 40 °C (lower energy)^59^. In contrast, a decrease in the peaks at 1604 cm^− 1^ and 1326 cm^− 1^ was observed in BF90. This may indicate the possible removal of extractives and lignin during drying at 90 °C. Similar results were reported by Da Costa et al.^60^ for the thermal treatment of açaí fibers, where the peaks decreased with increasing drying temperature (120 °C).

### Contact angle analysis

**Fig. 8:**
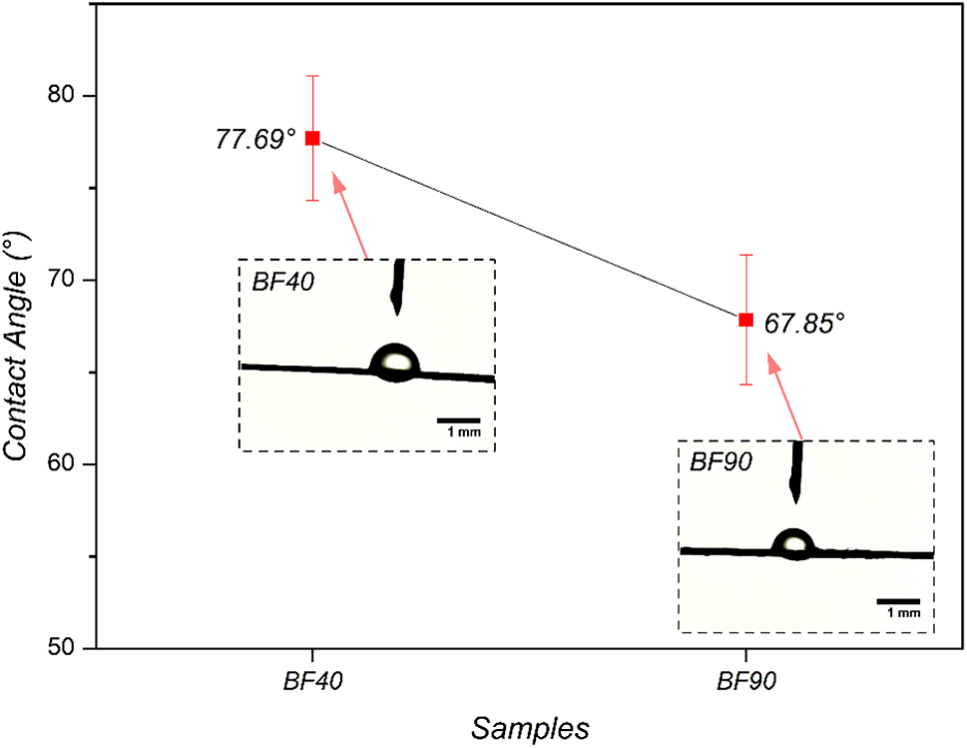
Measured contact angles of the dried BFs.

An effective indicator of the hydrophilicity of the fiber surface is the contact angle, as it is directly related to the surface free energy^61^. This can be used to determine the fiber wettability and predict its potential as a reinforcement in polymer matrices^62^. Figure 8 shows the measured contact angles of the dried BFs at 40 °C and 90 °C. The mean contact angle of BF40 was greater than that of BF90, which measured 77.69° and 67.85°, respectively. The difference in hydrophobicity between BF40 and BF90 may be attributed to the nonpolar materials, such as lignin and wax, present on the surface of BF40^62,63^. BF90 decreased the presence of these nonpolar materials, as shown by the FTIR and SEM analyses. This suggests that a higher drying temperature may increase the hydrophilicity of the BF. An improvement in the hydrophilicity of dried fibers at 90 °C could facilitate the use of coupling agents that act as mediators between the fibers and the polymer matrices. According to Elfaleh et al.^4^, coupling agents such as copolymer-anhydride can interact with the OH groups of cellulose and other groups that react with matrix molecules.

### TGA analysis

**Fig. 9:**
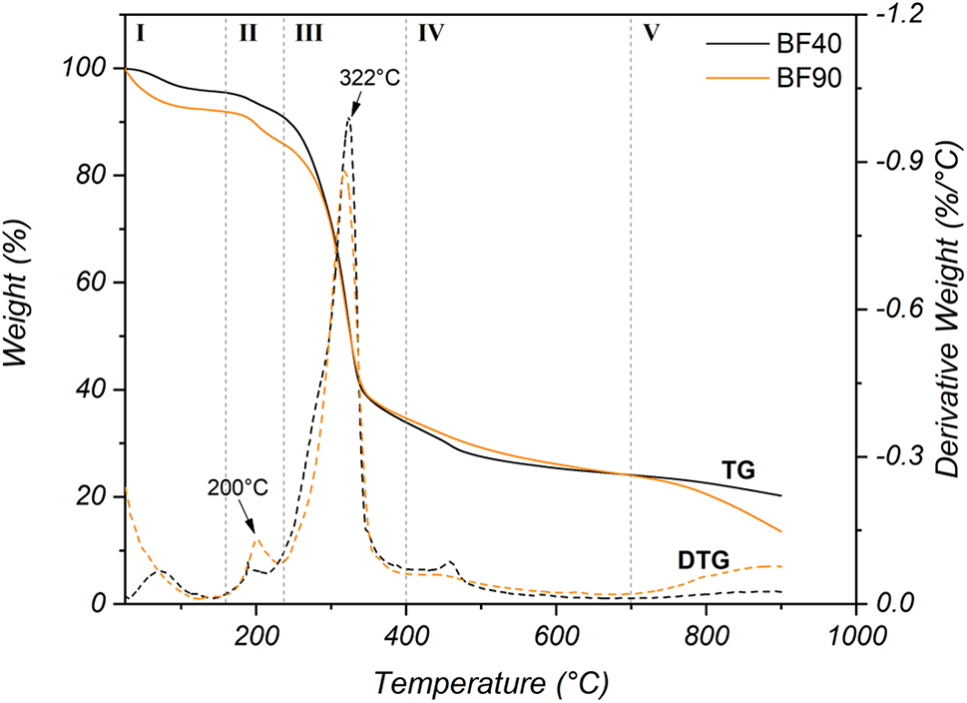
TG (solid line) and DTG (dashed line) curves of banana fibers dried at 40 °C and 90 °C.

The thermogravimetric (TG) curves and their respective first derivatives (DTG) of dry banana fiber samples are displayed in Fig. 9. TG curves reveal events of mass loss that occurred at different temperature ranges. Initially, mass loss was observed from 25 °C to 144 °C, corresponding to the evaporation of moisture from the samples and the loss of volatile compounds such as extractives^60^. A second event, marked by a shoulder in the DTG curves, was observed between 160 and 235 °C. This mass loss resulted in the onset of thermal degradation of hemicellulose^60,64^. It has been reported that the cleavage of some branched polysaccharides (such as xylose and mannose) and the decomposition of side chains in hemicellulose begin in this temperature range^64,65^. A third event can be observed in the TGA curves in the temperature range of 235–400 °C, with maximum mass losses of 58.1% and 51.8% for BF40 and BF90, respectively, corresponding to the thermal degradation of cellulose^66^. This is followed by a fourth mass loss event associated with lignin degradation, typically occurring at a relatively high temperature range of approximately 200–700 °C^5^. Residues such as ash are produced at 700 °C and above. The most significant differences between the two samples analyzed were observed in the first and second mass loss events. BF90 resulted in greater mass loss in the first event than did BF40. This could be related to the evaporation of free water that the fiber can absorb because of its relatively high degree of hydrophilicity^60,67^. This finding agrees with the results obtained via contact angle measurements. In addition, the DTG curve of the dried fibers at 90 °C showed a well-defined hemicellulose decomposition shoulder at approximately 200 °C, whereas in the BF40 sample, this peak was not as evident. Compared with BF90, the higher-temperature drying process likely volatilized some less stable lignin compounds, reducing the peak intensity associated with hemicellulose degradation in BF40^58^. This difference could be attributed to changes in the chemical composition of the fibers due to the higher drying temperature^7^. This is consistent with the FTIR results. Consequently, the change in the thermal stability of the samples at 700 °C can be attributed to the reduction in lignin content. The interaction between cellulose and lignin influences ash production during decomposition. A decrease in lignin content lowers the activation energy of the pyrolysis reactions in lignocellulosic fibers, thereby reducing the amount of residue^60^.

### Analysis of mechanical properties

**Fig. 10:**
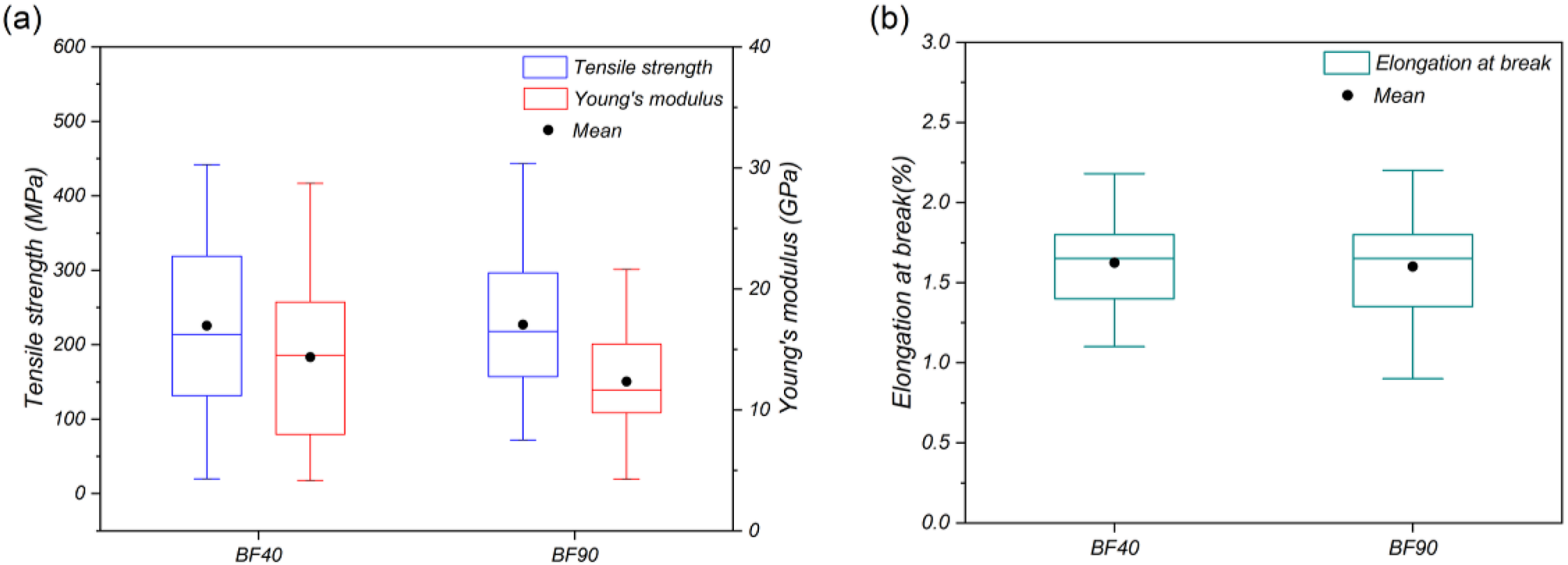
(**a**) Young’s modulus and tensile strength and (**b**) elongation at break of the dried fibers at 40 °C and 90 °C.

The tensile properties of BF40 and BF90 are shown in Fig. 10. BF40 had mean values of 225.58 ± 120.12 MPa for tensile strength and 14.35 ± 6.48 GPa for Young’s modulus, whereas BF90 had mean values of 226.95 ± 97.03 MPa and 12.34 ± 4.45 GPa, respectively. On the other hand, the mean elongation at break values for the BF40 and BF90 samples were 1.62 ± 0.29% and 1.60 ± 0.35%, respectively. These results are consistent with those of previous studies on banana pseudostem fibers^68,69^. Figure 10(a) shows no clear trend in the tensile strength and modulus of the banana fibers as a function of the drying temperature. Furthermore, it is also evident that there is considerable dispersion in the results for both samples. This can be attributed to the greater variability in fiber diameter due to the heterogeneous nature of the biological processes responsible for lignocellulosic fiber formation^70^, as discussed in subsection 3.5. Consequently, calculating the mechanical properties under the assumption of a fully circular fiber cross-section can lead to significant variability^47^. Like tensile strength and Young’s modulus, elongation at break does not clearly differ with respect to drying temperature. Therefore, drying did not affect banana fiber ductility. However, compared with other lignocellulosic fibers, such as coir, fique, and piassava fibers, BFs exhibit improved mechanical behavior, indicating that BFs are a potential reinforcement alternative for composite material manufacturing^71^.

**Fig. 11:**
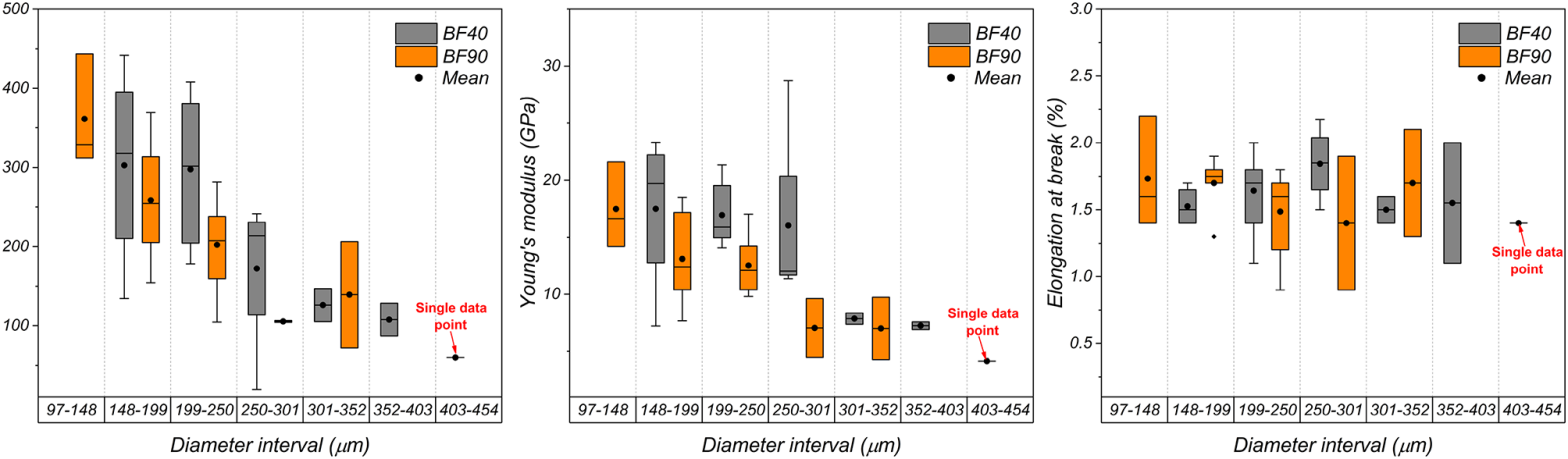
Variation in the tensile strength, Young’s modulus, and elongation at break with respect to the fiber diameter of the dried samples for each interval in Fig. 4.

Figure 11 displays the tensile properties of banana fibers dried at 40 °C and 90 °C for each diameter interval presented in Fig. 4. Mean tensile strength values between 400 MPa and 200 MPa were obtained for diameter intervals ranging from 97 to 250 μm. In addition, the mean Young’s modulus values within this range were between 10 GPa and 20 GPa. Moreover, the elongation at break of the samples in each diameter interval did not show a clear trend. These results show that the tensile strength and modulus tend to increase with decreasing fiber diameter. These results are consistent with those of Chaves et al.^33^, indicating that, in practice, thinner banana fibers are stiffer than thicker ones. The correlation between the fiber diameter and stiffness can be attributed to microstructural aspects such as the microfibril distribution^72^. During the drying process, the fibers undergo physical changes due to the removal of moisture and may experience a reduction in their outer volume^7^. Thinner fibers may have a more compact microfibril arrangement, contributing to a cross-section and fiber surface with fewer structural defects, resulting in a resistance region associated with a higher modulus. Conversely, thicker fibers may have a random distribution of a greater number of microfibrils, making them weaker than thinner fibers^72^. In this sense, when comparing the mechanical properties and fiber diameter distributions with respect to the drying temperature, the probability of having fibers with higher tensile strengths is within the diameter range of 148–250 μm. Therefore, the mechanical performance of BFs is not affected by drying at 40–90 °C. However, between the two samples, BF90 clearly has a lower mechanical response than BF40 in this diameter range. This may be related to the reduction in lignin in the fibers, a natural component that promotes adhesion between cellulose microfibrils, which are responsible for providing stiffness to the fibers^41,42^. In this way, drying at 40 °C may be considered a more effective pretreatment alternative in terms of mechanical properties.

## Conclusions

In this study, the influence of drying temperature on the properties of Colombian banana pseudostem fibers for their potential use as reinforcements in composite materials was investigated. The most highlighted conclusions obtained can be summarized as follows:

- The analysis of the drying curves revealed that the BF had a relatively high moisture removal rate at 90 °C. However, the moisture contents of BF40 and BF90 were similar, suggesting that drying at a higher temperature did not significantly impact the final moisture content.
- Morphological changes in the BF were observed via scanning electron microscopy (SEM). BF90 exhibited fewer impurities and smoother surface areas than BF40, which was attributed to the removal of noncellulosic materials at higher drying temperatures.
- FTIR analysis revealed vibrational modes related to the functional groups of the lignocellulosic materials, and mass loss events were observed in the TG curves. However, some minor differences were observed between BF40 and BF90, suggesting a decrease in the presence of lignin in the BF after drying at 90 °C. BF90 also showed a slight increase in hygroscopicity, as measured by the contact angle.
- The drying temperature did not influence the stiffness or ductility of the fibers, according to the analysis of the mechanical properties. However, a correlation between the fiber diameter and stiffness was observed. Thinner fibers within the diameter range of 148–250 μm presented increased tensile strength and Young’s modulus. This was attributed to the compact microfibril arrangement in the thinner fibers.
- Although the diameter values for BF90 tended to decrease due to physical changes caused by the removal of water and noncellulosic materials, in the range of 148–250 μm, BF40 presented a relatively high tensile strength and Young’s modulus. This is attributed to lignin reduction in the fibers, which favors adhesion between the cellulose microfibrils responsible for fiber stiffness. Consequently, drying at 40 °C is an effective pretreatment option for optimizing the mechanical properties of the BFs in composite applications. This approach has the potential to reduce both costs and energy consumption in the manufacturing of environmentally sustainable materials.

## Data Availability

The data related to this work can be obtained from the corresponding author by email request.

## References

[1] Akatwijuka, O. et al. Green hydrothermal extraction of banana cellulosic fibers by seawater-assisted media as an alternative to freshwater: Physico-chemical, morphological and mechanical properties. *Cellulose* **30**, 9989–10008 (2023).

[2] Mohammed, M. et al. Surface treatment to improve water repellence and compatibility of natural fiber with polymer matrix: Recent advancement. *Polym. Test.* **115**10.1016/j.polymertesting.2022.107707 (2022).

[3] Alomayri, T., Assaedi, H., Shaikh, F. U. A. & Low, I. M. Effect of water absorption on the mechanical properties of cotton fabric-reinforced geopolymer composites. *J. Asian Ceram. Soc.* **2**, 223–230 (2014).

[4] Elfaleh, I. et al. A comprehensive review of natural fibers and their composites: An eco-friendly alternative to conventional materials. *Rineng* **19**10.1016/j.rineng.2023.101271 (2023).

[5] de Oliveira, G. Q. et al. Drying of Banana pseudo-stem fibers: Evaluation of kinetic models, effective diffusivity, Thermodynamic Properties, and Structural characterization. *J. Nat. Fibers* **19**, 3654–3667 (2022).

[6] Adolfo, G. et al. Efecto De La Temperatura De Secado Sobre Las Propiedades Funcionales De La Fibra Dietaria Presente En La Citropulpa. *Rev. Lasallista Investig.* **7**, 2 (2010).

[7] Martinelli, F. R. B. et al. Influence of drying temperature on coconut-fibers. *Sci. Rep.* **14**10.1038/s41598-024-56596-z (2024). 10.1038/s41598-024-56596-z PMC10944830 38494529

[8] Baley, C., Le Duigou, A., Bourmaud, A. & Davies, P. Influence of drying on the mechanical behaviour of flax fibres and their unidirectional composites. *Compos. Part. Appl. Sci. Manuf.* **43**, 1226–1233 (2012).

[9] Azwa, Z. N., Yousif, B. F., Manalo, A. C. & Karunasena, W. A review on the degradability of polymeric composites based on natural fibres. *Mater. Des.* **47**, 424–442. 10.1016/j.matdes.2012.11.025 (2013).

[10] Väisänen, T., Haapala, A., Lappalainen, R. & Tomppo, L. Utilization of agricultural and forest industry waste and residues in natural fiber-polymer composites: A review. *Waste Manag. (Oxford)* **54**, 62–73. 10.1016/j.wasman.2016.04.037 (2016). 10.1016/j.wasman.2016.04.037 27184447

[11] Bai, Y. et al. Effects of different delignification and drying methods on Fiber properties of Moso Bamboo. *Polymer (Basel)* **14** (2022). 10.3390/polym14245464 PMC9783776 36559831

[12] Silva, N. G. S., Cortat, L. I. C. O. & Mulinari, D. R. Effect of alkaline treatment and coupling agent on thermal and mechanical properties of Macadamia Nutshell residues based PP composites. *J. Polym. Environ.* **29**, 3271–3287 (2021).

[13] Badanayak, P., Jose, S. & Bose, G. Banana pseudostem fiber: A critical review on fiber extraction, characterization, and surface modification. *J. Nat. Fibers* **20**10.1080/15440478.2023.2168821 (2023).

[14] FAO. Análisis del mercado del banano. Preprint at (2019). http://www.fao.org/3/ca9212es/ca9212es.pdf

[15] Twebaze, C. et al. Banana fiber degumming by alkali treatment and ultrasonic methods. *J. Nat. Fibers* **19**, 12911–12923 (2022).

[16] Ibarra, M. A. Márquez L. I. Identificación De usos potenciales para El Aprovechamiento De Los residuos generados en El proceso de beneficio del plátano (*Musa paradisiaca*) var. *Hartón Ingenierías* **9**, 2 (2022).

[17] Ministerio de Agricultura y Desarrollo Rural. Cadena de Plátano, Dirección de Cadenas Agrícolas y Forestales. Preprint at (2021). https://sioc.minagricultura.gov.co/Platano/Documentos/2021-03-31%20Cifras%20Sectoriales

[18] Cadena Ch, E. M., Vélez R, J. M., Santa, J. F. & Otálvaro, G. V. Natural fibers from plantain pseudostem (*Musa paradisiaca*) for use in fiber-reinforced composites. *J. Nat. Fibers* **14**, 678–690 (2017).

[19] Venegas, R. et al. Development and characterization of plantain (*Musa paradisiaca*) flour-based biopolymer films reinforced with plantain fibers. *Polym. (Basel)* 14 (2022). 10.3390/polym14040748 PMC8877579 35215661

[20] Oladele, I. O., Michael, O. S., Adediran, A. A., Balogun, O. P. & Ajagbe, F. O. Acetylation treatment for the batch processing of natural fibers: Effects on constituents, tensile properties and surface morphology of selected plant stem fibers. *Fibers* **8**, 1–19 (2020).

[21] Ernest, E. M. & Peter, A. C. Application of selected chemical modification agents on banana fibre for enhanced composite production. *Clean. Mater.* **5** (2022).

[22] Macedo, L. L., Vimercati, W. C., da Silva Araújo, C., Saraiva, S. H. & Teixeira, L. J. Q. Effect of drying air temperature on drying kinetics and physicochemical characteristics of dried banana. *J. Food Process. Eng.* **43** (2020).

[23] dos Santos, D. G., De Lima, A. G. B. & De Sousa Costa, P. The effect of the drying temperature on the moisture removal and mechanical properties of sisal fibers. *Defect Diffus. Forum* **380**, 66–71 (2017).

[24] Kamarudin, S. H. et al. A review on Natural Fiber Reinforced Polymer composites (NFRPC) for sustainable industrial applications. *Polymers* **14**, 3698 (2022). 36080773 10.3390/polym14173698 PMC9460194

[25] Norton, R. D. Colombia: Crop competitiveness by region evaluated via tracks 1 and 2. In *The Competitiveness of Tropical Agriculture* (ed. Norton, R. D.), 231–305 (Academic Press, 2017). 10.1016/B978-0-12-805312-6.00010-6

[26] International Plant Names Index (IPNI). *Musa paradisiaca* L. IPNI & Retrieved August 19, from (2024). https://www.ipni.org/n/797595-1

[27] Instituto Amazónico de Investigaciones Científicas SINCHI. Ficha de Especímenes – 8066. Herbario Virtual COAH & Retrieved August 19, from (2024). https://herbario.sinchi.org.co/herbario_v/especimenes/ficha/8066/

[28] Kemp, I. C. et al. Methods for processing experimental drying kinetics data. *Dry. Technol.* **19**, 15–34 (2001).

[29] Onwude, D. I., Hashim, N., Janius, R. B., Nawi, N. M. & Abdan, K. Modeling the thin-layer drying of fruits and vegetables: A review. *Compr. Rev. Food Sci. Food Saf.* **15**, 599–618 (2016). 33401820 10.1111/1541-4337.12196

[30] Beigi, S., Sobati, M. A. & Charkhi, A. Drying kinetics of thorium oxalate: Experimental investigation and modeling. *Prog Nucl. Energy* **88**, 240–244 (2016).

[31] Hall, M. B. & Mertens, D. R. Comparison of alternative neutral detergent fiber methods to the AOAC definitive method. *J. Dairy. Sci.* **106**, 5364–5378 (2023). 37331877 10.3168/jds.2022-22847

[32] Paternina Reyes, M. J., Silgado, U., Santa, J., Marín, J. F. & Lopera, C. H. A. & Espitia Sanjuán, L. A. Cashew Nutshells: A promising filler for 3D Printing filaments. *Polym. (Basel)* **15** (2023). 10.3390/polym15224347 PMC10674345 38006072

[33] Chaves, Y. S. et al. Evaluation of the density, mechanical, thermal and chemical properties of babassu fibers (Attalea speciosa.) For potential composite reinforcement. *J. Mater. Res. Technol.* **23**, 2089–2100 (2023).

[34] Han, W., Shin, J. & Ho Shin, J. Low-cost, open-source contact angle analyzer using a mobile phone, commercial tripods and 3D printed parts. *HardwareX* **12**, e00327 (2022). 10.1016/j.ohx.2022.e00327 PMC9272025 35833036

[35] Temitayo Oyewo, A., Olugbemiga Oluwole, O., Olufemi Ajide, O., Emmanuel Omoniyi, T. & Hussain, M. Banana pseudo stem fiber, hybrid composites and applications: A review. *Hybrid. Adv.* **4**, 100101 (2023).

[36] Soraisham, L. D., Gogoi, N., Mishra, L. & Basu, G. Extraction and evaluation of properties of Indian Banana Fibre (Musa Domestica Var. Balbisiana, BB Group) and its processing with ramie. *J. Nat. Fibers* **19**, 5839–5850 (2022).

[37] Patel, B. Y. & Patel, H. K. Retting of banana pseudostem fibre using Bacillus strains to get excellent mechanical properties as biomaterial in textile & fiber industry. *Heliyon* **8** (2022). 10.1016/j.heliyon.2022.e10652 PMC9494229 36158073

[38] Silva, F. S., Ribeiro, C. E. G., Demartini, T. J. da C., & Rodríguez, R. J. S. Physical, chemical, mechanical, and microstructural characterization of banana pseudostem fibers from musa sapientum. *Macromol. Symp.* 394 (2020).

[39] Kandemir, A., Pozegic, T. R., Hamerton, I., Eichhorn, S. J. & Longana, M. L. Characterisation of natural fibres for sustainable discontinuous fibre composite materials. *Materials* **13** (2020). 10.3390/ma13092129 PMC7254363 32375396

[40] Ribeiro, B. M. et al. Chemical treatment of banana tree pseudostem particles aiming the production of particleboards. *Ciênc Agrotec.* **38**, 1 (2014).

[41] Gupta, U. S. et al. Surface modification of banana fiber: A review. *Mater. Today Proc.* **43**, 904–915 (2020).

[42] Pereira Junio, R. F. et al. Thermochemical and structural characterization of promising carnauba novel leaf fiber (Copernicia prunifera). *J. Mater. Res. Technol.* **18**, 4714–4723 (2022).

[43] Cecci, R. R. R., Passos, A. A., de Aguiar Neto, T. C. & Silva, L. A. Banana pseudostem fibers characterization and comparison with reported data on jute and sisal fibers. *SN Appl. Sci.* **2** (2020).

[44] Karuppuchamy, A. K., R. & R., S. Novel banana core stem fiber from agricultural biomass for lightweight textile applications. *Ind. Crops Prod.* **209** (2024).

[45] Alwani, M. S. et al. Microstructural study, tensile properties, and scanning electron microscopy fractography failure analysis of various agricultural residue fibers. *J. Nat. Fibers* **12**, 154–168 (2015).

[46] Mumthas, A. C. S. I., Wickramasinghe, G. L. D. & Gunasekera, U. S. W. Effect of physical, chemical and biological extraction methods on the physical behaviour of banana pseudo-stem fibres: Based on fibres extracted from five common Sri Lankan cultivars. *J. Eng. Fiber Fabr.* **14** (2019).

[47] Barrera-Fajardo, I., Rivero-Romero, O. & Unfried-Silgado, J. Investigation of the effect of chemical treatment on the properties of colombian banana and coir fibers and their adhesion behavior on polylactic acid and unsaturated polyester matrices. *Fibers* 12 (2024).

[48] Jaramillo-Quiceno, N., Vélez, R., Ch, J. M. C., Restrepo-Osorio, E. M., Santa, J. F. & A. & Improvement of mechanical properties of pineapple leaf fibers by mercerization process. *Fibers Polym.* **19**, 2604–2611 (2018).

[49] Thomas, S., Paul, S. A., Pothan, L. A. & Deepa, B. *Natural Fibres: Structure, Properties and Applications. In Cellulose Fibers: Bio- and Nano-Polymer Composites 3–42* (Springer Berlin Heidelberg, 2011). 10.1007/978-3-642-17370-7_1

[50] Zuluaga, R. et al. Cellulose microfibrils from banana rachis: Effect of alkaline treatments on structural and morphological features. *Carbohydr. Polym.* **76**, 51–59 (2009).

[51] Gañán, P., Zuluaga, R., Velez, J. M. & Mondragon, I. Biological natural retting for determining the hierarchical structuration of banana fibers. *Macromol. Biosci.* **4**, 978–983 (2004). 15497200 10.1002/mabi.200400041

[52] Fávaro, S. L., Ganzerli, T. A., de Carvalho Neto, A. G. V., da Silva, O. R. R. F. & Radovanovic, E. Chemical, morphological and mechanical analysis of sisal fiber-reinforced recycled high-density polyethylene composites. *Express Polym. Lett.* **4**, 465–473 (2010).

[53] Shruthi, S. & Vishalakshi, B. Development of banana pseudostem supported polymeric adsorbent for effective removal of cationic dyes from wastewater. *Fibers Polym.* **25**, 1765. 10.1007/s12221-024-00608-2 (2024).

[54] Wang, C. et al. Investigation into the correlation between the chemical structure of lignin and its temperature-dependent pyrolytic product evolution. *Fuel* **329** (2022).

[55] Li, M., Wei, T., Qian, C. & Liang, Z. Preparation of microcrystalline cellulose from Rabdosia rubescens residue and study on its membrane properties. *Sci. Rep.* **11** (2021). 10.1038/s41598-021-98645-x PMC8460781 34556803

[56] Ru, S., Yang, R., Li, X. & Yang, S. Effect of the interfacial bonding performance between coir fiber and epoxy resin matrix by adding adhesive layer. *Fibers Polym.*10.1007/s12221-024-00640-2 (2024).

[57] Horikawa, Y. et al. Prediction of lignin contents from Infrared Spectroscopy: Chemical Digestion and Lignin/Biomass ratios of Cryptomeria japonica. *Appl. Biochem. Biotechnol.* **188**, 1066–1076 (2019). 30783948 10.1007/s12010-019-02965-8

[58] Liebl, G. F. et al. Study of drying of banana pseudo-stem and influence of particle sizes on biomass saccharification and cellulosic ethanol production. *Bioenergy Res.* **12**, 605–625 (2019).

[59] Salem, K. S., Naithani, V., Jameel, H., Lucia, L. & Pal, L. A systematic examination of the dynamics of water-cellulose interactions on capillary force-induced fiber collapse. *Carbohydr. Polym.* **295** (2022). 10.1016/j.carbpol.2022.119856 35989003

[60] Tavares, D. C. F. F. et al. Thermal treatment of açaí (Euterpe oleracea) fiber for composite reinforcement. *Polimeros* **30** (2020).

[61] Long, Y., Zhang, Z., Fu, K. & Li, Y. Efficient plant fibre yarn pre-treatment for 3D printed continuous flax fibre/poly(lactic) acid composites. *Compos. B Eng.* **227** (2021).

[62] Raharjo, W. W., Soenoko, R., Irawan, Y. S. & Suprapto, A. The influence of chemical treatments on cantala fiber properties and interfacial bonding of cantala fiber/recycled high density polyethylene (rHDPE). *J. Nat. Fibers* **15**, 98–111 (2018).

[63] Vijay, R. et al. Characterization of raw and alkali treated new natural cellulosic fibers from Tridax procumbens. *Int. J. Biol. Macromol.* **125**, 99–108 (2019). 30528990 10.1016/j.ijbiomac.2018.12.056

[64] Faleeva, Y. M., Lavrenov, V. A. & Zaichenko, V. M. Investigation of plant biomass two-stage pyrolysis based on three major components: Cellulose, hemicellulose, and lignin. *Biomass Convers. Biorefin.* **14**, 14519–14529 (2022).

[65] Reis, R. S., Tienne, L. G. P., Souza, D., de Marques, H. S., Monteiro, S. & F. V. & N. characterization of coffee parchment and innovative steam explosion treatment to obtain microfibrillated cellulose as potential composite reinforcement. *J. Mater. Res. Technol.* **9**, 9412–9421 (2020).

[66] Bhunia, A. K., Mondal, D., Parui, S. M. & Mondal, A. K. Characterization of a new natural novel lignocellulose fiber resource from the stem of Cyperus platystylis R.Br. *Sci. Rep.* **13** (2023). 10.1038/s41598-023-35888-w PMC10272156 37322033

[67] Wang, Q., Xiao, S. & Shi, S. Q. The effect of hemicellulose content on mechanical strength, thermal stability, and water resistance of cellulose-rich fiber material from poplar. *BioRes* **14**, 3 (2019).

[68] Xu, S., Xiong, C., Tan, W. & Zhang, Y. Microstructural, thermal, and tensile characterization of banana pseudo-stem fibers obtained with mechanical, chemical, and enzyme extraction. *BioResources* **10**, 2 (2015).

[69] Idicula, M., Malhotra, S. K., Joseph, K. & Thomas, S. Dynamic mechanical analysis of randomly oriented intimately mixed short banana/sisal hybrid fibre reinforced polyester composites. *Compos. Sci. Technol.* **65**, 1077–1087 (2005).

[70] Monteiro, S. N., Margem, F. M., Braga, F. O., da Luz, F. S. & Simonassi, N. T. Weibull analysis of the tensile strength dependence with fiber diameter of giant bamboo. *J. Mater. Res. Technol.* **6**, 317–322 (2017).

[71] Srinivas, K., Lakshumu Naidu, A. & Bahubalendruni, M. V. A. R. A review on chemical and mechanical properties of natural fiber reinforced polymer composites. *Int. J. Perform. Eng.* **13**, 2 (2017).

[72] Teles, M. C. A. et al. Fique fiber tensile elastic modulus dependence with diameter using the weibull statistical analysis. *Mater. Res.* **18** (2015).

